# Tricuspid leaflet autotransplantation for mitral annular enlargement in biventricular repair

**DOI:** 10.1016/j.xjtc.2025.10.016

**Published:** 2025-11-01

**Authors:** Hüseyin Sicim, Reena M. Ghosh, Angelo Giannopoulos, Kandice Mah, Pedro J. Del Nido

**Affiliations:** a Department of Cardiac Surgery, Boston Children's Hospital, Harvard Medical School, Boston, Mass; b Department of Cardiology, Boston Children's Hospital, Harvard Medical School, Boston, Mass

**Figure:**
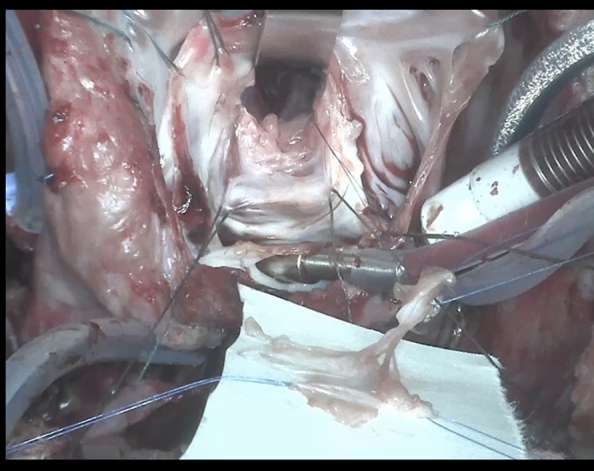
Partially resected inferior tricuspid leaflet prepared for autologous transplantation to the mitral position.

**Central Message**

Autologous transplantation of the tricuspid leaflet offers a feasible and durable surgical option to augment the mitral valve in complex congenital heart disease with severe mitral annular hypoplasia.

Reconstructive strategies for congenital mitral stenosis in the setting of left ventricular hypoplasia and complex intracardiac anatomy remain surgically challenging.^1^ In particular, patients with double-outlet right ventricle (DORV) and concomitant mitral hypoplasia often present with limited options for achieving biventricular circulation. Although mitral valvuloplasty and endocardial fibroelastosis resection can improve inflow dynamics, annular and leaflet deficiencies may preclude adequate valve function.^2^ We present a novel surgical approach involving partial tricuspid valve autotransplantation to enlarge the mitral annulus and augment leaflet coaptation during biventricular repair in a patient with severe hypoplastic mitral valve. Written patient informed consent was obtained for the publication of their data; institutional review board approval was not required.

## Case Description

A 15-month-old boy presented with a history of complex congenital heart disease, including {S,D,D} DORV, conoventricular and muscular ventricular septal defects (VSDs), pulmonary stenosis, and congenital mitral stenosis presented for biventricular repair. The patient had previously undergone biventricular staging with mitral valve repair, endocardial fibroelastosis resection, and muscular VSD closure at 6 months of age. Follow-up transthoracic echocardiography revealed a severely hypoplastic effective orifice area of the mitral valve (0.17 cm^2^), mitral valve annulus lateral diameter 0.50 cm (z score −6.0), mean gradient 9 mm Hg, a large conoventricular VSD with predominant right-to-left shunting, an overriding aorta with preserved function, mild tricuspid regurgitation, and moderate pulmonary stenosis. In addition, a 3-dimensional cardiac computed tomography model demonstrated the severely hypoplastic effective orifice area of the mitral valve. The left ventricle was apex-forming but hypoplastic (indexed left ventricular end-diastolic volume, 39 mL/m^2^). Total pulmonary vascular resistance index was 1.5 WU·m^2^. The test occlusion of the atrial septum resulted in an increase in mean left atrial pressure from 16 mm Hg to 32 mm Hg, and mitral inflow gradient increased to 16 mm Hg, indicating persistent mitral stenosis. On the basis of these findings, the patient was taken to the operating room for complete biventricular repair.

## Operative Technique and Findings

The anterior commissure of the mitral valve was completely fused, and the leaflets were significantly thickened. Sharp dissection was used to thin the anterior and posterior leaflets, including the annulus, allowing removal of excessive tissue that was reducing the annular diameter. Both leaflets remained considerably thickened; therefore, further thinning was performed. Despite increased leaflet mobility postthinning, the annulus remained relatively small, and it was deemed unlikely that a satisfactory repair could be accomplished using the 2 native leaflet tissues alone. Notably, the tricuspid valve was markedly enlarged compared with normal. Upon inspection, the inferior leaflet of the tricuspid valve—measuring approximately 12 mm at its base—was harvested to augment the mitral valve, specifically targeting the A1, A2, P1, and P2 segments (Video 1).

The inferior tricuspid leaflet was carefully dissected from its hinge point, and the head of the associated papillary muscle was excised. Attention was then redirected to the mitral valve, where the papillary muscles corresponding to the inferior mitral leaflet were identified alongside an optimal site for attachment of the new tricuspid leaflet on the posterior wall near the expected location of the A2 papillary muscle. The papillary muscle head was reattached to the posterior wall near the anterior papillary muscle insertion using 7-0 Gore-Tex sutures with pledgets. Additional chordae supporting the tricuspid valve were sutured onto the head of the inferior papillary muscle to provide support for the new leaflet.

The new leaflet was then secured to the mitral annulus with a running 6-0 PROLENE suture (Ethicon) extending from A1 to P2. The side-to-side anastomosis of the new leaflet to the native mitral leaflets was performed with interrupted 7-0 PROLENE sutures. This reconstruction substantially improved coaptation and resulted in a competent orifice that comfortably accommodated a 10- to 11-mm sizer.

Further dissection was undertaken in the early annular region to widen the area, where native leaflets exhibited better mobility and good coaptation—consistent with preoperative echocardiographic findings identifying this region as having the greatest native leaflet opening. Valve testing demonstrated satisfactory coaptation at this stage. The VSD was then approached via the partially mobilized tricuspid valve. A Dacron patch was used to baffle flow from the left ventricle to the aorta and was secured circumferentially with 5-0 PROLENE sutures.

Subsequent evaluation of the tricuspid valve revealed an inferior gap. The anterior leaflet was detached up to approximately the 12:00-o'clock position anteriorly, along with part of the inferior leaflet extending just anterior to the conduction tissue, allowing advancement of these 2 leaflets to reduce the annulus concurrently. The advanced leaflets were stabilized and reattached to the annulus with a 2-layer 6-0 PROLENE suture technique, effectively reducing the annular size. Subsequently, the septal leaflet and a portion of the anterior leaflet were approximated in a side-to-side fashion using interrupted 7-0 PROLENE sutures to enhance coaptation and valve competence. Valve testing confirmed good coaptation. Finally, the interatrial septal defect was repaired using a pulmonary homograft patch, secured in place with running 6-0 PROLENE sutures.

During the course of stay in the intensive care unit, although the patient was extubated, and transthoracic echocardiography performed in the first postoperative week revealed severe tricuspid regurgitation and moderate mitral regurgitation. Because of the suspicion of possible dehiscence, the patient was taken back to the operating room. Reoperation was performed for tricuspid valve repair with annular plication at the site of dehiscence of the leaflets with augmentation using an autologous pericardial patch, and mitral valve repair with closure of a small annular dehiscence at the base of the transposed leaflet. The patient was successfully extubated on postoperative day 3, and transthoracic echocardiography performed on the same day revealed trivial tricuspid regurgitation and mild mitral regurgitation. At the third postoperative month, follow-up transthoracic echocardiography demonstrated mild-to-moderate mitral regurgitation and stenosis, accompanied by mild tricuspid stenosis and regurgitation. Although these early postoperative results appear favorable, extended follow-up is warranted to more accurately determine the long-term durability and functional efficacy of the surgical repair.

## Discussion

Mitral stenosis presents a significant challenge in patients undergoing biventricular repair, particularly in the context of complex congenital heart disease such as DORV with a hypoplastic left ventricle.^3^ The patient's mitral valve was characterized by extreme annular hypoplasia (z score −6.0) and fused anterior commissure, contributing to a severely restricted effective orifice area and clinically significant inflow obstruction. Traditional leaflet thinning and commissurotomy alone were insufficient to restore adequate valve function, necessitating innovative surgical strategies.^4^

Autologous leaflet transplantation, in this case harvesting the inferior leaflet of the tricuspid valve, served as an effective technique for annular augmentation and enhancement of leaflet coaptation. This approach offers several advantages. First, it avoids the use of prosthetic material in the left heart, thereby reducing risks of infection, thrombosis, and potential calcification. Second, it uses viable native tissue with preserved chordal and papillary support, which may confer improved long-term durability and valve dynamics.

Finally, the concurrent tricuspid valve repair ensured preservation of right atrioventricular valve competence, addressing the balance of biventricular function. The leaflet autotransplantation application in the setting of congenital mitral stenosis with severe annular hypoplasia remains limited. This report adds to the growing body of evidence supporting innovative autologous tissue use to overcome anatomical constraints in pediatric valve repair.

## Conclusions

The autologous inferior tricuspid leaflet transplantation represents a viable and innovative surgical option for mitral valve repair in patients with severe annular hypoplasia and complex congenital heart disease, facilitating effective biventricular repair with favorable early valve function.

## Conflict of Interest Statement

The authors reported no conflicts of interest.

The *Journal* policy requires editors and reviewers to disclose conflicts of interest and to decline handling or reviewing manuscripts for which they may have a conflict of interest. The editors and reviewers of this article have no conflicts of interest.

## Supplementary Data

Video 1 Surgical video of tricuspid leaflet autotransplantation for mitral annular enlargement in biventricular repair. Video available at: https://www.jtcvs.org/article/S2666-2507(25)00476-6/fulltext.
