# Supplementary figures and images for: Tricuspid leaflet autotransplantation for mitral annular enlargement in biventricular repair
Source: JTCVS Tech. 2025 Nov 1;35:102141. doi: 10.1016/j.xjtc.2025.10.016 (PMC12881805; doi:10.1016/j.xjtc.2025.10.016)

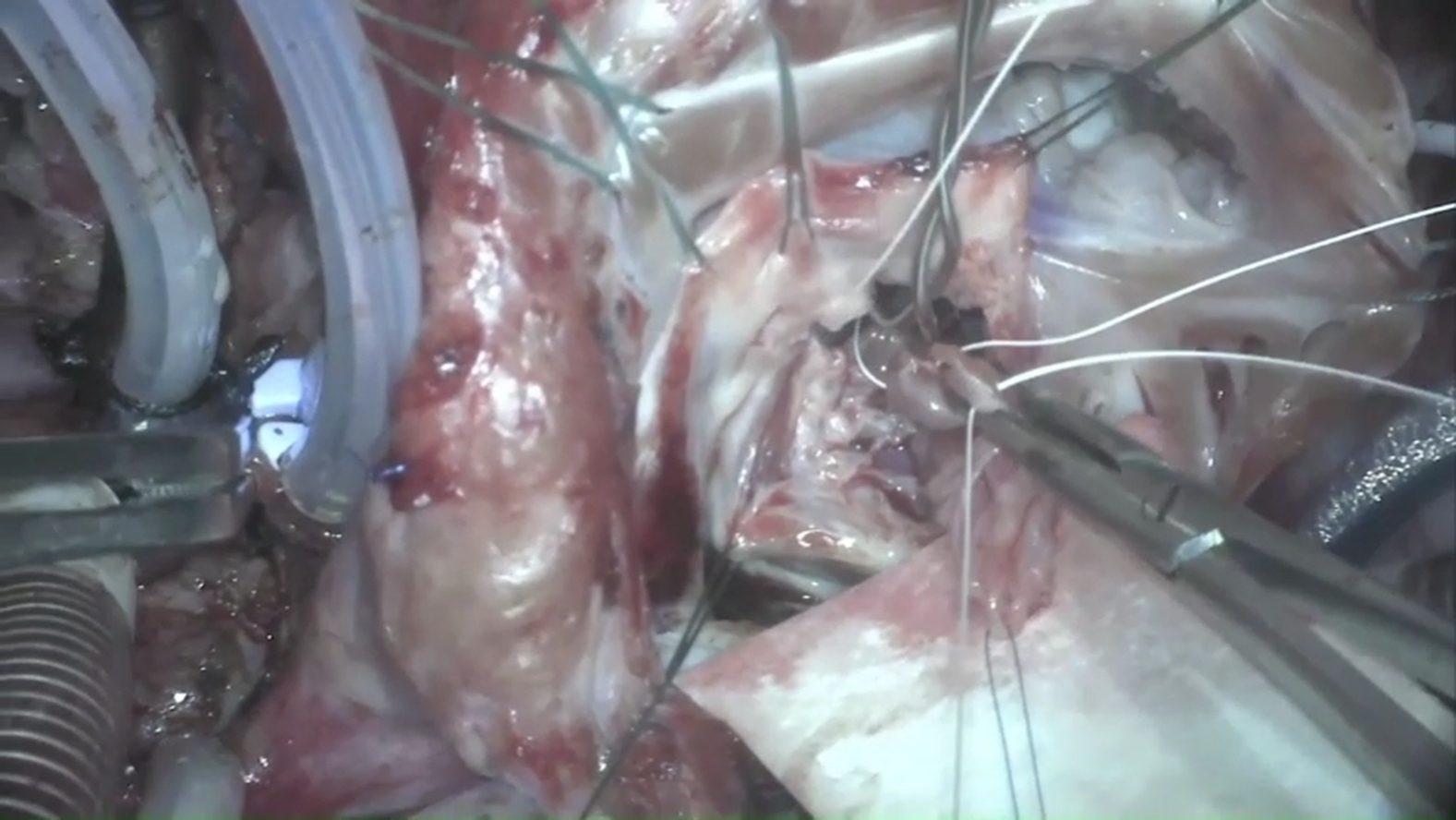

Supplement: Video 1 — Surgical video of tricuspid leaflet autotransplantation for mitral annular enlargement in biventricular repair. Video available at: https://www.jtcvs.org/article/S2666-2507(25)00476-6/fulltext. [file fx2.jpg]
